# Mucins as Diagnostic and Prognostic Biomarkers in a Fish-Parasite Model: Transcriptional and Functional Analysis

**DOI:** 10.1371/journal.pone.0065457

**Published:** 2013-06-12

**Authors:** Jaume Pérez-Sánchez, Itziar Estensoro, María José Redondo, Josep Alvar Calduch-Giner, Sadasivam Kaushik, Ariadna Sitjà-Bobadilla

**Affiliations:** 1 Nutrigenomics and Fish Growth Endocrinology Group, Instituto de Acuicultura Torre de la Sal (IATS-CSIC), Castellón, Spain; 2 Fish Pathology Group, Instituto de Acuicultura Torre de la Sal (IATS-CSIC), Castellón, Spain; 3 INRA, UR1067 NuMeA Nutrition, Metabolism Aquaculture, Saint Pée-sur Nivelle, France; Inserm, France

## Abstract

Mucins are *O*-glycosylated glycoproteins present on the apex of all wet-surfaced epithelia with a well-defined expression pattern, which is disrupted in response to a wide range of injuries or challenges. The aim of this study was to identify mucin gene sequences of gilthead sea bream (GSB), to determine its pattern of distribution in fish tissues and to analyse their transcriptional regulation by dietary and pathogenic factors. Exhaustive search of fish mucins was done in GSB after *de novo* assembly of next-generation sequencing data hosted in the IATS transcriptome database (www.nutrigroup-iats.org/seabreamdb). Six sequences, three categorized as putative membrane-bound mucins and three putative secreted-gel forming mucins, were identified. The transcriptional tissue screening revealed that Muc18 was the predominant mucin in skin, gills and stomach of GSB. In contrast, Muc19 was mostly found in the oesophagus and Muc13 was along the entire intestinal tract, although the posterior intestine exhibited a differential pattern with a high expression of an isoform that does not share a clear orthologous in mammals. This mucin was annotated as intestinal mucin (I-Muc). Its RNA expression was highly regulated by the nutritional background, whereas the other mucins, including Muc2 and Muc2-like, were expressed more constitutively and did not respond to high replacement of fish oil (FO) by vegetable oils (VO) in plant protein-based diets. After challenge with the intestinal parasite *Enteromyxum leei*, the expression of a number of mucins was decreased mainly in the posterior intestine of infected fish. But, interestingly, the highest down-regulation was observed for the I-Muc. Overall, the magnitude of the changes reflected the intensity and progression of the infection, making mucins and I-Muc, in particular, reliable markers of prognostic and diagnostic value of fish intestinal health.

## Introduction

Mucins belong to a heterogeneous family of high molecular weight proteins composed of a long peptidic chain with a large number of tandem repeats that form the so-called mucin domain. These repeats are particularly rich in serine, threonine and proline residues (the PTS domain). The PTS domain is extensively *O*-glycosylated through GalNAc at the Ser and Thr residues, and account for 50–80% of the mass of the molecule [1]. These PTS regions differ in size and sequence from one mucin to another and are not conserved between species and within species [2].

There are two structurally distinct families of mucins: large secreted gel forming (SGFM) and membrane-bound forms [3]. SGFM include MUC2, MUC5AC, MUC5B, MUC6 and MUC19. Their N-terminal and C-terminal regions flanking the PTS domain code for cysteine-enriched domains similar to the pro-von Willebrand factor (pro-vWF). The N-termini contain vW type D (vW-D) domains, Cys-rich C8 domains (C8) and the C-termini contain cystine-knot (CK) domains. The CK domain is also found in other secreted proteins such as the NDP (Norries Disease Protein). Many SGFM also contain multiple copies of a “naked” cysteine-enriched domain (CYS domain) that interrupt or are adjacent to the PTS domain. Most of these two types of cysteine-enriched domains contribute to mucin oligomerization by disulphide bonding and are highly conserved, which implies an important common function in many different organisms and therefore, inter-species comparisons of the these domains are useful for analysing mucins during evolution [4], [5]. By contrast, membrane-bound mucins (MUC1, MUC3, MUC4, MUC12, MUC13, MUC14, MUC15, MUC16, MUC17 and MUC18) have a single membrane-spanning region anchored to the plasmalema and *O*-glycosylated PTS ectodomains that form rod-like structures that extend over 100 nm from the cell surface [6]. They also have typically an extracellular highly conserved SEA domain (domain first found in *S*ea urchin sperm protein, *E*nterokinase and *A*grin) that resides between the PTS and the transmembrane (TM) domains, with some exceptions, such as MUC4/Muc4 that lacks a SEA domain and instead has other three domains (NIDO,AMOP, vWD) that are not found in other membrane-bound mucins [5], [7], [8]. The available information indicates that SGFM appeared earlier in metazoan evolution, and the appearance of a TM component provided an additional level of defence to promote the growth, repair and survival of epithelial cells [9]. Hence, these two main classes of mucins have both unique and shared structural features, which serve to protect the underlying epithelia against a wide range of injuries (bacteria, virus, parasites, toxins, pH, etc.). This protection leads to coordinate cell proliferation, differentiation and apoptosis among other cellular responses [10], [11]. It is not surprising, thereby, that mucins stay under intensive investigation as highly promising biomarkers and therapeutic targets in cancer and inflammatory diseases [12], [13], [14].

Thus far, more than 20 mucin genes have been identified and characterized in higher vertebrates, but several mucins are likely waiting for discovery due to the technical problems associated to the large size and repetitive sequences of the mucin chain-peptide. Recently, it has become apparent that sequence databases can be useful tools to find new candidate genes. A better understanding of the molecular identity and functional regulation of mucins is, thereby, mandatory to assign specific roles to a given mucin gene or isoform within and among different vertebrate species. This is especially relevant in the case of lower vertebrates and fish in particular. Thus, the first goal of the present study was to provide a comprehensive overview of the mucin gene family through searches in the updated cDNA repository database (http://www.nutrigroup-iats.org/seabreamdb) of gilthead sea bream (GSB) (*Sparus aurata*) [15], a perciform fish extensively cultured in the Mediterranean basin. The second goal was to underline the tissue-specific expression pattern of GSB mucins in skin, gills and the gastrointestinal tract. The third goal was to determine whether these mucins were altered by nutritional and pathogen challenges. To pursue this issue, the myxozoan parasite *Enteromyxum leei* was used as an intestinal infection model. This parasite causes severe desquamative enteritis, cachexia and eventually death [16]. Thus far there are no preventive or curative treatments for this enteromyxosis, although growth, histopathological and genome wide-gene expression criteria have highlighted that the disease outcome is worse and faster when fish are fed vegetable oils (VO) rather than fish oil (FO) as the most important source of dietary oils [17], [18]. In a previous study of the mucosal carbohydrate pattern of the intestine of GSB, the VO diet produced a significant decrease of goblet cells (mucins secreting cells) with neutral and acidic mucins in the anterior intestine and middle intestine, and also of those with carboxylic mucins and sialic acid in the middle intestine. In addition, *E. leei* infection had a strong depletion effect on the number of goblet cells, which was stronger in VO-fed fish [19]. Thus, our experimental hypothesis is to assess if this different health phenotype is explained, at least in part, by different nutritionally-mediated effects on the intestine-mucin gene expression pattern and regulation.

## Materials and Methods

### Molecular Identity and Structure Analysis

The recently updated GSB cDNA transcriptome database (http://www.nutrigroup-iats.org/seabreamdb) was used to identify mucin-encoding genes. First, the database was term-searched for automatically annotated mucin genes. In a second step, mucin-encoding genes were identified by BLAST queries using mucin-sequence predictions derived from genome sequencing of tilapia and fish model species. When multiple GSB sequences were identified, they were manually curated for frame-shifting errors and a PCR approach was used to confirm that the construct belonged to the same gene transcript.

For structure analysis, the edited sequences were blasted against the SMART database in the normal SMART mode, searching for Pfam domains and internal repeats. Transmembrane segments were predicted by the TMHMM2 server and those of mucin type GalNAc O-glycosylation sites by NetOGlyc 3.1 server.

### Animal Care, Experimental Design and Sample Collection

Juveniles of GSB were reared in the indoor experimental facilities of the Institute of Aquaculture Torre de la Sal (IATS-CSIC). Day length and temperature followed natural changes at our latitude (40°5′N; 0°10′E), except during the infection trials when water was temporarily heated to keep temperature always above 18°C. The oxygen content of water was always higher than 85% saturation, and unionized ammonia remained below toxic levels (<0.02 mg/l). Except when indicated, fish were fed a commercial diet (Proaqua, Palencia, Spain) containing 47% protein and 21% lipid.

A first approach for tissue screening of mucin gene expression was carried out in one year-old GSB (n = 10) with 150 g average body weight. Fish were randomly selected from rearing tanks of stock animals and target tissues (skin, gills, oesophagus, stomach, anterior (AI), middle (MI) and posterior (PI) intestine were taken for gene expression study.

To analyse the effect of the parasite infection and nutritional condition alone or in combination on mucin gene expression, two different experimental trials were undertaken in which naïve pathogen-free GSB were challenged with *E. leei* by two different routes. In the first trial, the infection was performed by anal intubation as previously described [20]. Briefly, 20 GSB (average initial weight = 127.5 g) were intubated with 1 ml of *E. leei* infected-intestinal scrapings (recipient fish, RCPT) and control fish (CTRL, average initial weight = 133.5 g) were intubated with the same volume of PBS. After 40 days post intubation (p.i.) 7 fish from both groups were killed for parasite diagnosis and samples of AI, MI and PI were taken for mucin gene expression studies. In the second trial, the infection was performed by exposure to *E. leei*-contaminated effluent, as previously published [17]. Briefly, GSB were fed during 9 months either a FO diet or a blend of VO at 66% of replacement (66 VO diet) (Table S1). After this period, fish from both diet groups (initial body weight = 224 g) were exposed to *E. leei*-effluent (RCPT) or kept unexposed (CTRL). After 102 days post exposure (p.e.), fish were sacrificed for parasite diagnose and only samples of PI were collected for gene expression analysis in view of the results obtained in the first trial.

In both infection trials, fish were kept in 5 µm-filtered and UV-irradiated sea water (37.5 ‰ salinity), the mean water temperature during the challenges was about 21°C. Parasite diagnosis was performed in intestine samples fixed in 10% buffered formalin processed following routine histological procedures and embedded in paraffin or resin. The final prevalence of infection was 92.9% in trial 1, and 73.3% in R-FO and 93.3% in R-66 VO in trial 2.

In all experiments, target tissues were rapidly excised, frozen in liquid nitrogen in less than 10 min, and stored at −80°C until RNA extraction and gene expression analysis.

### Ethics Statement

All experiments were carried out in accordance with the principles published in the European animal directive (86/609/EEC) for the protection of experimental animals and in accordance with national (Royal Decree RD1201/2005) laws for the protection of animals used in scientific experiments, and approved by the Consejo Superior de Investigaciones Científicas (CSIC) ethics committee and IATS Review Board, with permits associated to project AGL2009-13282-C02-01. In all lethal samplings, fish were overnight fasted and decapitated under benzocaine anesthesia (3-aminobenzoic acid ethyl ester, 100 mg/l) (Sigma, St. Louis, MO, USA), and all efforts were made to minimize suffering.

### RNA Extraction and RT Procedure

Total RNA from target tissues was isolated by means of the Ambion MagMax-96 for Microarray kit (Applied Biosystems) after tissue homogenization in TRI reagent at a concentration of 100 mg/ml following the manufacturers’ instructions. RNA quantity and purity was determined by Nanodrop (Thermo Scientific) with absorbance ratios at 260 nm/280 nm above 1.9. Synthesis of cDNA was performed with the High-Capacity cDNA Archive Kit (Applied Biosystems) using random decamers and 500 ng total RNA in a final volume of 100 µl. Reverse transcriptase (RT) reactions were incubated 10 min at 25°C and 2 h at 37°C. Negative control reactions were run without RT.

### Gene Expression Analyses

Quantitative real-time PCR was performed using an iCycler IQ Real-time Detection System (Bio-Rad, Hercules, CA, USA) as described elsewhere [21]. Briefly, diluted RT reactions were used for PCR reactions in 25 µl volume. Each PCR-well contained a SYBR Green Master Mix (Bio-Rad) and specific primers at a final concentration of 0.9 µM were used to obtain amplicons of 50–150 bp in length (Table 1). The efficiency of PCR reactions varied between 90% and 99% and the specificity of reaction was verified by analysis of melting curves, serial dilutions of RT reactions, and electrophoresis and sequencing of PCR amplified products. Reactions were performed in triplicate and the fluorescence data acquired during the extension phase were normalized by the delta-delta method using β-actin as housekeeping gene [22]. Four genes (β-actin, elongation factor 1, α-tubulin and 18S rRNA) were tested for stability using the GeNorm software. The most stable reference gene in relation to dietary treatment and crowding exposure was β-actin (M score = 0.21), and it was used in the normalization procedure.

**Table 1:** Forward and reverse primers for real-time PCR.

Gene name	Symbol	Accession number		Primer sequence
Intestinal mucin	I-Muc	JQ27712	F	GTG TGA CCT CTT CCG TTA
			R	GCA ATG ACA GCA ATG ACA
Mucin 2	Muc2	JQ27710	F	ACG CTT CAG CAA TCG CAC CAT
			R	CCA CAA CCA CAC TCC TCC ACA T
Mucin 2-like	Muc2-like	JQ27711	F	GTG TGT GGC TGT GTT CCT TGC TTT GT
			R	GCG AAC CAG TCT GGC TTG GAC ATC A
Mucin 13	Muc13	JQ27713	F	TTC AAA CCC GTG TGG TCC AG
			R	GCA CAA GCA GAC ATA GTT CGG ATA T
Mucin 18	Muc18	JQ27714	F	ATG GAG GAC AGA GTG GAG G
			R	CGA CAC CTT CAG CCG ATG
Mucin 19	Muc19	JQ27715	F	TGC TTG CTG ATG ACA CAT
			R	TTC ACA TAG GTC CAG ATA TTG A

### Phylogenetic Analysis

Multiple sequence alignments were carried out with ClustalW and a phylogenetic tree was constructed on the basis of amino acid differences (poisson correction) with the Neighbour Joining (NJ) algorithm (complete deletion) in MEGA version 5.0 [23]. A total of 20 mucin sequences from 8 species were used in the analysis. Reliability of the tree was assessed by bootstrapping, using 1000 bootstrap replications.

### Statistical Analysis

Data on gene expression are represented as the mean ± SEM of 6–8 fish. For each mucin gene, the specific effect of tissue, pathogen exposure and dietary treatment on mucin mRNA levels were analyzed by Student t-test (when two groups were compared) or by one-way analyses of variance (ANOVA-I) followed by Student-Newman-Keuls test. When the test of normality or equal variance failed, a Mann-Whitney Rank Sum test or a Kruskal-Wallis ANOVA-I on ranks followed by Dunn’s method was applied instead, respectively. The significance level was set at P<0.05. All the statistical analyses were performed using Sigma Stat software (SPSS Inc., Chicago, IL, USA).

## Results

### Structure and Phylogenetic Analyses of Mucin Gene Candidates

Searches in the GSB database recognized (E-value ≤1e-33) three contigs of 121–449 clones in depth with complete codifying sequences of 736 (Muc2), 434 (Muc13) and 643 (Muc18) amino acids in length (Table 2). Three additional non-overlapping contigs of 16–73 clones in depth and 1674–1849 bp in length were identified as partial-mucin mRNA sequences and annotated as intestinal mucin (I-Muc) (E-value 5e-33), Muc2-like (E-value 0) and Muc19 (E-value 0). These new GSB sequences were uploaded in GenBank with accession numbers JQ277712 (I-Muc), JQ277710 (Muc2), JQ277711 (Muc2-like), JQ277713 (Muc13), JQ277714 (Muc18) and JQ277715 (Muc19).

**Table 2:** Classification of identified genes according to BLAST searches.

Contig	F^a^	Size (nt)	Annotation^b^		Best match^c^	E^d^	CDS^e^
C2_11326	73	1849	I-Muc	XP_002937513		5e-33	<1–1020
C2_3396	337	2798	Muc2	XP_002667589		0	453–2663
C2_22932	16	1469	Muc2-like	CAF91948		0	<1–>1469
C2_1615	449	2421	Muc13	XP_002661255		1e-33	81–1385
C2_4523	121	3929	Muc18	XP_003450918		0	336–2267
C2_28812	24	1674	Muc19	XP_003445129		0	<1–1268

a Number of sequences.

b Gene identity determined through BLAST searches.

c Best BLAST-X protein sequence match.

d Expectation value.

e Codifying sequence.

**Figure 1:**
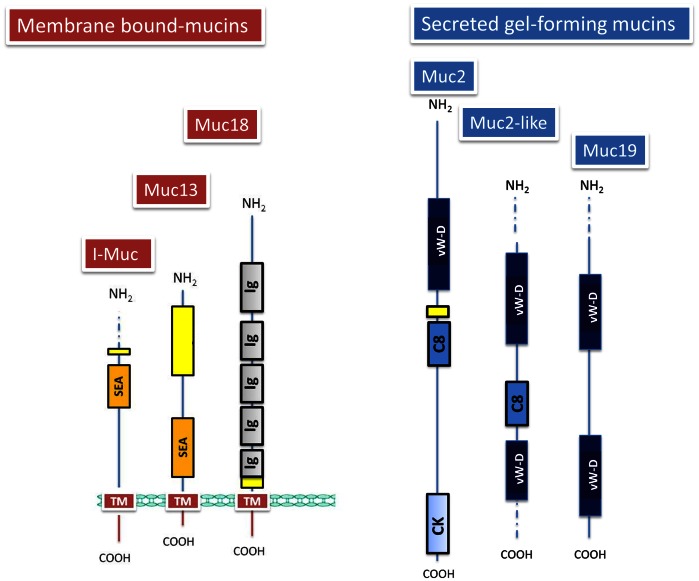
Schematic representation of the molecular structure of the six gilthead sea bream mucins. Various functional domains are indicated in boxes: *O*-glycosylated region or PTS domain (yellow), extracellular proteolytic cleavage site SEA domain (orange), transmembrane domain (TM) (red), immunoglobulin domain (Ig) (grey), vW-D domain (dark blue), C8 domain (blue), and cystine knot domain (CK) (light blue). Discontinuous lines at NH_2_ or COOH ends represent the predicted size of the lacking sequences in partial proteins according to homology comparisons.

As depicted in Figure 1, the sequences annotated as I-Muc, Muc13 and Muc18 share the characteristic TM domain of the membrane-bound mucin subclass with a cytoplasmic tail of 26–52 amino acids in length and a strict conservation in the case of I-Muc and Muc13 of an extracellular proteolytic cleavage site (SEA domain) next to the TM domain. The sequence recognized as Muc18, also called CD146 or melanoma cell adhesion molecule (Mel-CAM), possesses a large number of immunoglobulin domains through the entire extracellular region, and is at the edge between mucin and mucin-like molecules that are qualified as endothelial and leucocyte mucins. The sequences annotated as Muc2, Muc2-like and Muc19 are unequivocally within the subclass of SGFM, typically characterized by the presence of a large number of cysteine-rich domains, such as C8, CK and vW-D domains, but we could not identify PTS domains in Muc2-like and Muc19. Figure S1 shows the deduced amino acid sequence of the reported GSB mucins together with sequence and domain alignments with orthologs from other species.

**Figure 2:**
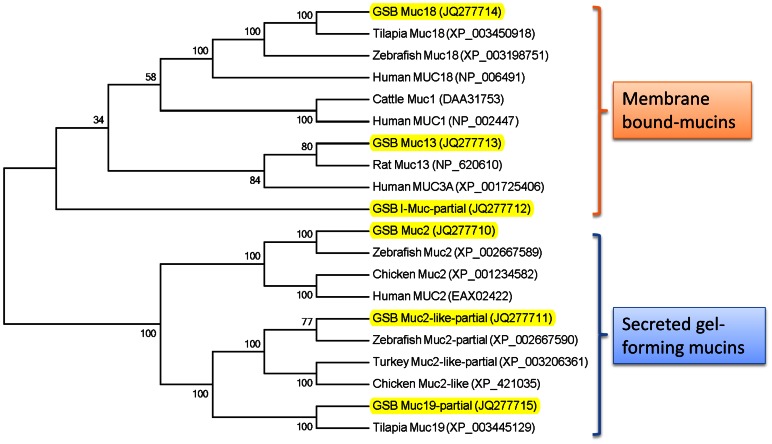
Phylogenetic tree of membrane-bound and secreted gel-forming mucins. Gilthead sea bream mucins are highlighted in yellow. GenBank accession numbers are provided for each sequence.

The phylogenetic tree undertaken for GSB mucins evidenced two major clades (membrane-bound mucins and SGFM) according to the present hierarchy of vertebrates (Figure 2). Of note, within the long-branch covering the membrane-bound mucins, the node of Muc18 is related to neighbouring Muc1 node rather than to cluster of Muc13 and the I-Muc outlier. Conversely, the nodes of Muc2, Muc2-like and Muc19 appear as monophyletic groups within the cluster of SGFM.

### Gene Expression Analysis

**Figure 3:**
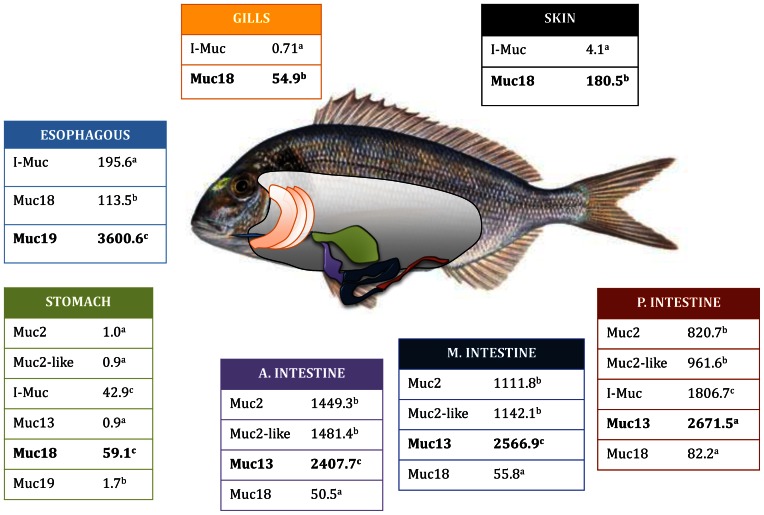
Relative mRNA expression of gilthead sea bream mucins in different tissues. For each tissue, the most abundant mucin is in bold face and different superscript letters stand for statistically significant differences (P<0.05) between mucins.

The mucin gene expression pattern was tissue-specific in GSB with a relatively low expression level in skin, gills and stomach (Figure 3). Overall, Muc18 and I-Muc were expressed constitutively, whereas Muc19 was predominantly detected at very high levels in the oesophagus. Likewise, Muc13 was mostly represented in the intestinal tissue, with an antero-posterior increasing profile, whereas Muc2 and Muc2-like, also highly expressed, had an opposite gradient (postero-anterior). By contrast, the contig annotated as I-Muc was differentially expressed across the intestine with high levels at the posterior segment and was non-detectable in the other two intestinal segments. Detailed expression values of all the mucin genes for all the studied tissues are reported in Table S2.

**Figure 4:**
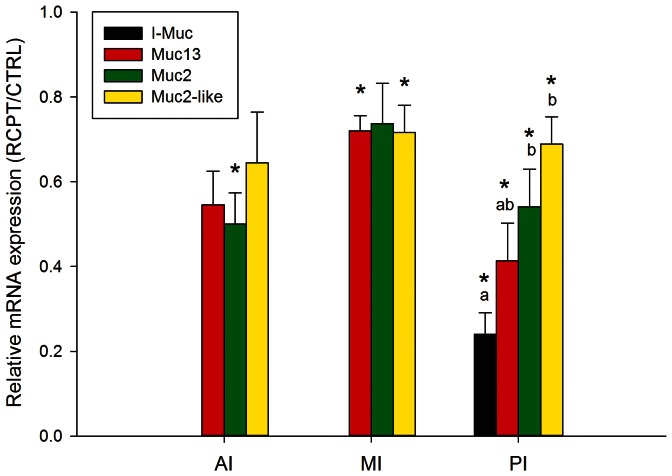
Relative mRNA expression levels of mucins in the anterior (AI), middle (MI) and posterior (PI) intestinal segments of gilthead sea bream infected by *Enteromyxum leei* (Trial 1). Each bar represents the mean ± SEM of 7 infected animals. Asterisks indicate statistically significant differences (P<0.05) with control (CTRL) fish. Different letters stand for statistically significant differences (P<0.05) between mucins within each intestinal segment.

**Figure 5:**
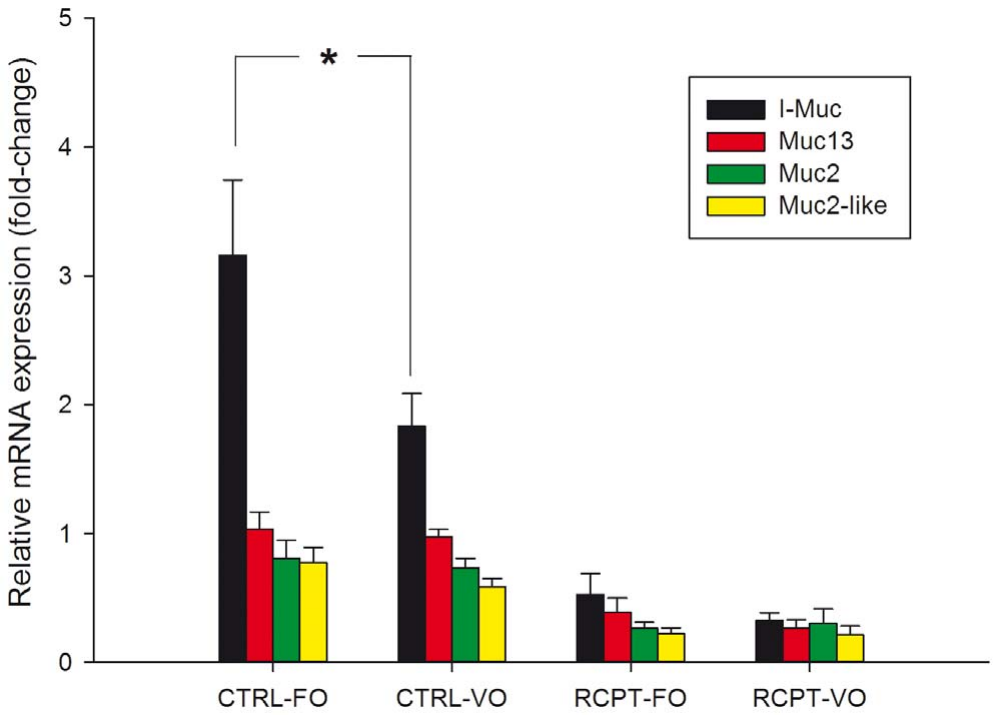
Relative mRNA expression levels of mucins in the posterior intestine of gilthead sea bream fed vegetable oils (VO) or fish oil (FO) diets and infected by *Enteromyxum leei* (RCPT) or kept unexposed to the parasite (CTRL) (Trial 2). Each bar represents the mean ± the SEM of 6–10 animals. Asterisk indicate statistically significant differences (P<0.05) between CTRL fish fed different diets. Significant differences were also found between each CTRL group and its corresponding RCPT group for the four mucins (not indicated to avoid confusion in symbol interpretations).

Parasitic infection also induced changes in mucin gene expression, as fish infected by anal intubation with *E. leei* shared an overall decrease in mucin gene mRNA levels that was especially evident at the PI (Figure 4). At this intestine segment, the disruption of the gene expression pattern was significant for the four studied mucins, though the down-regulation of the intestinal mucin was higher than those of Muc2 and Muc2-like, with intermediate values for Muc13. The same results were achieved when fish with a different nutritional history were challenged by water effluent with *E. leei* (Figure 5). Of note, a diet effect (FO diet vs. VO diet) on the mucin gene expression was not found for Muc2, Muc2-like and Muc13 in either control fish or infected fish, but the expression level of the I-Muc in fish not exposed to parasite infection was significantly lower in fish fed the VO diet than in fish fed the FO diet. When comparing each challenged diet group with their corresponding control group, again the four studied mucins were also significantly down-regulated.

## Discussion

Mucins, both secreted and membrane-bound, are multifunctional glycoproteins that contribute to the protective mucus gel layer either directly or through their ectodomains. They were thought to exclusively protect and lubricate epithelial surfaces, but recent molecular biology studies indicate that some mucins are additionally involved in signalling pathways that lead to coordinated cellular responses such as cell proliferation, differentiation and adhesion, immune response, apoptosis, bacterial adhesion/inhibition and secretion of specialized cellular products. Their pattern of distribution in human tissues and organs is well known, but its knowledge in lower vertebrates is just starting to be elucidated. Furthermore, the aberrant expression of mucins or their alterations in glycosylation are well documented in a variety of inflammatory or malignant human diseases [24], making them valuable markers to distinguish between normal and disease conditions. In fact, many mucins are used as prognostic and diagnostic markers in malignant diseases involving epithelial cells [25], [26]. In most fish studies, immunocytochemical, cytochemical and biochemical techniques have been applied to determine the effect of environmental pollutants and pathogens on mucins and mucin producing cells (goblet cells, GC) [27], [28], [29], [30]. However, fish mucin gene expression studies are very scarce in part due to the limitations imposed by the size and nature of the sequence of mucin genes. Thus, this is the first study which analyses in depth the gene expression profile of six mucins in fish tissues and how they are affected by nutritional and pathological challenges.

First of all, it is noteworthy that the molecular identity of mucins categorized as SGFM (Muc2, Muc2-like and Muc19) was unequivocally established on the basis of Blast searches (E-value = 0) and phylogenetic analysis of the GSB sequences annotated in our transcriptome database as complete or almost complete codifying sequences. More uncertain is the molecular identity of the mucins categorized as membrane-bound mucins, but even in this case no doubt exists for the annotated Muc18 given its particular structural feature and the high amino acid identity with the best matches corresponding to genome sequence predictions of tilapia (*Oreochromis niloticus*) and zebrafish (*Danio rerio*). Nevertheless, a number of mucin mRNAs are higher than 10 kbp and contain large repetitive units, which poses a challenge towards new gene discovery and annotation as pointed out by Micallef et al. [31] when they explored the skin transcriptome of Atlantic salmon. These authors indicated that several salmon isotigs exhibited homology to mammalian mucins (MUC2, MUC5AC and MUC5B), but definitive conclusions were not drawn until the open reading frames were entirely sequenced. In our case, the sequence annotated as Muc13 shows a relatively low level of amino acid identity with mammalian orthologues, but the open reading frame is completely sequenced and its molecular identity is unambiguous, regardless of its relatively low level of conservation through vertebrate evolution. However, in the case of I-Muc, there is not a clear orthologue in mammals and it is difficult to establish its precise molecular identity in the absence of a reference genome, but intriguingly it shared a tissue-specific gene expression pattern with a high abundance at PI. This lack of a true orthologue is, however, not surprising since *in silico* analysis in puffer fish (*Fugu rubripes*) suggested that the number of SGFM has been conserved through the evolution of vertebrates, whereas the family of transmembrane mucins is markedly expanded [32].

When analysing the tissue-specific gene expression of membrane-bound mucins in GSB a very different pattern was found for each of them. Muc18, though constitutively found in all studied organs, was the most abundant mucin in gills and skin. Interestingly, in humans, the expression of Muc18 in normal adult tissues appears limited to endothelial cells in vascular tissue throughout the body, and it has been proposed as a biomarker for prognosis in cutaneous melanoma [33], [34]. The deduced amino acid sequence indicates that Muc18 is a member of the immunoglobulin superfamily and shows the greatest sequence similarity to a group of neural cell adhesion molecules expressed during organogenesis. In agreement with this, it has been speculated that MUC18 may also be developmentally regulated and mediates intercellular adhesion. This adhesion is supposed to be particularly relevant in fish skin and gills directly exposed to the turbulences of the water, as they are the major barriers to the aquatic environment, and play a crucial role in protection against pathogens together with numerous other biological processes, such as osmoregulation and ion exchange.

Another membrane-bound mucin gene candidate, the so-called I-Muc was constitutively expressed in all the studied organs except at AI and MI, but it was mostly expressed at PI and more importantly, it was highly regulated by the nutritional background and by *E. leei* infection. Previous histochemical analyses did not reveal statistically significant differences between the three intestinal segments in the same CTRL animals for any of the studied mucins (neutral, acidic, sialomucins). However, the VO diet induced a significant decrease of GC with neutral and acidic mucins in the AI and MI, and also of those with carboxylic mucins and sialic acid in the MI in CTRL fish [19], but not in PI. Therefore, with the study of the expression levels, we went further in the mucin analysis and were able to detect a mucin (intestinal mucin) that is clearly down-regulated both by the diet and by the infection at PI. Finally, Muc13 had an antero-posterior increasing trend, similar to the increasing expression pattern from small intestine to rectum in humans [35]. MUC13 is expressed abundantly by colorectal [36], ovarian [37] and gastric [38] human cancers, and is considered an early marker for cancer screening [39]. The down-regulation of Muc13 in infected GSB, particularly at the PI, is in agreement with the significant reduction of GC positive for sialic acid in early infected fish and the fact that it was the most reduced type of GC in fish with a high intensity of infection [19], since Muc13 is the predominant sialomucin. Furthermore, this lack of regulation could contribute to the negative inflammatory effects of the enteromyxosis, since a protective role for Muc13 in the colonic murine epithelium has been shown [40].

The analyses of the gene expression pattern of SGFM showed that Muc19 was by far the highest expressed mucin, present predominantly in the oesophagus and scarcely in the stomach of GSB. This mucin is one of the major components of salivary gland secretions in humans as its expression is very high in mucous cells of the submandibular gland, and it is also present in the tracheal epithelium [41]. As true salivary glands are not found in fish [42], the mucins produced in the oesophagus could be homologous to those of the saliva of terrestrial animals and contribute to the digestion of food. Further studies involving also the oral cavity of different fish species with different food and feeding habits may shed light to the possible adaptive modifications of these oesophagic mucins. Other SGFM such as Muc2 and Muc2-like were the predominant mucins in the whole intestinal tract of GSB, together with the aforementioned Muc13. The profile of these three mucins was down-regulated in the three intestinal segments of parasitized GSB, which was more pronounced and significant for all of them at the PI (trial 1). In trial 2, this down-regulation at the PI was confirmed in RCPT fish, regardless of the diet, but no effect of the diet was found in CTRL fish. This is in accordance with previous results using cytochemistry, in which the strongest reduction of GC positive for different types of mucins was observed at the PI of *E. leei*-infected fish [19].

Muc2 and Muc2-like had a postero-anterior gradient. Similarly, Muc2 is known to show a preferential expression in the small intestine of sheep [43]. However, in common carp, Muc2 gene expression was higher in the second intestinal segment that in the first one [44]. In humans and mice, Muc2 is the predominant mucin produced by intestinal GC. In addition, Muc2 also has a function as a tumour suppressor [26], [45]. Furthermore, its expression is decreased in patients with ulcerative colitis and collective data supports a model in which Muc2 is essential for the protection of the intestinal epithelium against commensal bacteria and potential pathogens in mice [24].

Mucin expression in other enteric pathogen models has been reported to be regulated in different ways depending on the type of pathogenicity [46], [47]. In most nematode infections, GC are increased and the expression of some mucins is enhanced, causing thickening of the glycocalyx and changes in the glycosylation that may help to expel the parasites [43], [48], [49]. Nevertheless, GC reduction as in the current study has also been reported in *Echinostoma caproni* infections [50] and in clinically important enteric pathogens, such as *Shigella* [51], [52], *Campylobacter* [53] and *Citrobacter rodentium* [54]. In fish-parasite models, there is no information on the effects of pathogens on mucin gene expression, but only on the changes in the number and type of GC cells as a consequence of infection [55], [56], [57], [58], [59]. In *E. leei*-infected GSB, the altered intestinal mucus secretion provoked a reduction of microbial adhesion [29], but further studies are necessary to understand the modifications of the complex intestinal microbial balance.

This is the first report on the effect of the diet on the gene expression of several mucins in fish. The only remarkable previous study has shown an increased Muc5B expression in the skin of common carp fed β-glucan, but no significant changes were found for Muc2 [44]. In humans and other animal models, certain dietary components, such as fiber and probiotics can influence mucin secretions [60], [61]. In particular, short-chain fatty acids, such as butyrate [62], [63], certain probiotics [64], glucans [65] and food-derived peptides [66] stimulated the gene expression of several mucins, whereas other phytochemicals such as resveratrol [67] and quercitin [68] down-regulated the expression of Muc5AC.

*De novo* synthesis of mucins is controlled primary at the transcriptional or post-transcriptional level and a large number of biologically active molecules have been shown to regulate mucin synthesis [69], [70], [71], [72], [73], [74]. In our fish model, we can only speculate about the possible regulation by some immune factors that indeed have been described to be altered by enteromyxosis, such as the down-regulation of some cytokines in chronic infections [75]. Responsiveness to these cytokines provides a link between mucins, innate mucosal immunity, and mucosal inflammatory responses [76]. In addition, several plant products included in fish diets have been reported to modulate both innate and adaptive immune responses of fish [77], and particularly in GSB [78], [79]. This study has analysed just a few factors that regulate intestinal mucins and much more work is still needed to understand its molecular signalling and their ontology.

In conclusion, since the intestine plays an important role in the ingestion and absorption of nutrients, and is the barrier to the entrance of microbes and microbial products, the disregulation of mucins may endanger its functional integrity. Therefore, the intestinal mucins described in the present study could serve as prognostic markers of an intestinal phenotype susceptible to dietary changes and also as diagnostic markers of the pathological effects of intestinal pathogens involving a GC depletion phenotype. Further immunohistochemical and/or *in situ* hybridisation studies will help to confirm and localize this quantitative differential expression in the fish tissues.

## Supporting Information

Figure S1 **Deduced amino acid sequences of GSB mucins and alignment with mucin orthologs.**
(PDF)

Table S1 **Fish Oil (FO) and 66% Vegetable Oil (66** **VO) diet ingredients.**
(DOCX)

Table S2 **Relative expression values of gilthead sea bream mucins in all studied tissues.**
(DOCX)
