# Supplementary material for: Mucins as Diagnostic and Prognostic Biomarkers in a Fish-Parasite Model: Transcriptional and Functional Analysis
Source: PLoS One. 2013 Jun 12;8(6):e65457. doi: 10.1371/journal.pone.0065457 (PMC3680472; doi:10.1371/journal.pone.0065457)
Supplement: Figure S1 — Deduced amino acid sequences of GSB mucins and alignment with mucin orthologs. (PDF) [file pone.0065457.s001.pdf]

Figure S1. Deduced amino acid sequences of GSB mucins and alignment with mucin orthologs. Black boxes mark the deduced initiation and termination codons. Functional domains are indicated in coloured boxes: *O*-glycosylated region or PTS domain (yellow), extracellular proteolytic cleavage site SEA domain (orange), transmembrane domain (TM) (red), immunoglobulin domain (Ig) (grey), vW-D domain (dark blue), C8 domain (blue), and cysteine knot domain (CK) (light blue). In each amino acid alignment, conserved residues with the GSB sequence are represented by dots.

Intestinal mucin:

```
GSB          : MTAPTTPHQCTEKDCPGCVGTCIFNTTLGGCFCNCEEFAFGDACVFGENDTSADIDPENRPTRNATFTLRILMDFDAAFE
GSB          : DINSPQSLEFIKTLMQELEALCKEADPQNFKTVQVIKLSSGSVLAESVVDYLYANNETQIQFVNNQLDGVLTKILNDTSN
GSB          : LNKISQVFNASVTLKELTFEPPEITNITDLKPFVNCTKFAQYTAEVINGQWQCVGPCKTNPDYCHQHGECFNNIKEGPVC
GSB          : RCFETSLKQYYGQQCDLFRYGPGFYGALFGSLAGALLLVIMIVIAVIAKKRYMGVWKTTNPYNRRLSAFDEEFFDFSETG
GSB          : DHNFGAAGTYTSQGSRQHL
```

Mucin 2:

```
GSB       : MARCIENNTVEIIPYECPPIQNITCANGKKPVILYDEYHCCNYSACDCECKGWGDPHYITFDGSYYSYQGNCTYILMEEI
Zebrafish : ........I................E...N.......HY..Q.YT...F.E............L................
Chicken   : N.I.......V...VI.E.PPKP..S..LA..Q.I.DDL..WHWE...Y.T............L................
Human     : ..T.KY.......KV..E.PPMP..S..LQ..R.E.PDG..WHWE...Y.T............L................

GSB       : SPRK-HLTIYIDNVLCDPTEDVSCPRSIIISYDSQVITLMNHNLIGAVQLEALKDGVRLKLPYSQGDVEVLNSGLNLIFE
Zebrafish : R.QY-.........F...I.H............N.....K...FF.GA.....MGD.L.T...AHNG.R..S.S...FLS
Chicken   : EK.VDNFG.....YH..ARDI.....A...RHE..E.R..TVKPN-T....VTV.KQP.A...KKFG.S.YE....R.V.
Human     : ..SVDNFG.....YH...NDK.........RHE..E.L.KTVH..-P....VQV.RQA.A...KKYG...YQ....Y.VD

GSB       : IPRLNVIITFGINGFSVSLPFEHFGNNTQGHCGTCNNNQADDCMLPGGQLVESCAVMADYWPAKHINPPDCKIPTLLPTS
Zebrafish : ..Q...D....AT....N....................K.........I..DD.S......A.SG..DEM.TP..A...V
Chicken   : ..E.K.N...NGLS...R...SL.......Q..I....T....R..N.N.A.N.ET.....QVVDPSK.Q.SPGL.PTKA
Human     : ..E.G.....NGLS...R...HR.....K.Q....T.TTS......S....SN.EAA..Q.LVNDPSK.H.PHS.STTKR

GSB       : APEPPP------TVTPCKPDSICDMLKSSVFEECHPFVSPDRFFQGCVFDSCHVSNAEVECTSLQTYAAACAQEGICLHW
Zebrafish : GGDIK.------.PK...AHPD.F..G.E...A..AH...EN..M..E........PA.V........RS.S.L......
Chicken   : PSTTTG--------K...ES...E..WG....K.REV.K.....AA......T.P.LD.......I...I..D.N...D.
Human     : PAVTV.GGGKTTPHKD.T.SP..Q...D...A...AL.P.QH..DA......F.PGSS...A...A...L....N...D.

GSB       : RNHTK-LCTSDCPSDKVYKPCGPAEQPTCDDNFDKPPSKFTTEGCFCPDGMKLFNQESGICVDKCGCIDPEGIPREFNER
Zebrafish : ..Y.N-..NIE.AE....N......P.....IPEQRTITVP.......E.TM...KD.....S......AS.T......V
Chicken   : .S..NG...YE..KH.E..A...I..T..KSSPQNET.IKQI........TM...SGVD....T....GLDM.....G..
Human     : ....HGA.LVE...H.E.QA.........KSSSSQQNNTVLV......E.TMN.APGFD...KT....G.DN.....G.H

GSB       : FEYNCQDCICEESTKTVTCKPKVCPAPPQTNCTGPGFVLVSQTNPSDPCCSVFVCQCHSNTCPETNMNCPIGYKPVFSVP
Zebrafish : ......E....RA.........S..GGNPEV..E......NY....E....KQ..N.DVSM..PL.KK.N...A..LD..
Chicken   : .TA.......L.GGNG.V.E.HK..EQNKKS...K..YE..VV.SE....PTVT.K.N.SL.TTEPPK.T...EVFSY..
Human     : .....K....L.GGSG.I.Q..R.SQK.V.H.VED.TY.A..V..A.T..N.T..K.N.SL.K.KPSV.....EVKSK.V

GSB       : EGGCCPEHRCDPKRVCVHKDIEYQPGMSVPAAVCQDCTCSFEVDPKSGLFKITCRLHQCKS-CDLGYEYVQTHSDECCGK
Zebrafish : ..K....I..E.........T.....T...VID..E.K...D.....QF...K.GFV..NEK..P.....DDF.-D....
Chicken   : SDE...VY..V.......Q.A..L.NS..FVDK.H..F..N...IS.Q.NV...ERIP.N.Y.EP...LQHVQG-.....
Human     : P.R...FYW.ES.G....G.A.....SP.YSSK....V..DK..NN.L.NV.A.THVP.N.S.SP...L..APG-...K.

GSB       : CVQTHCMVN-VNGTKQLVALGKTWSPLGSNCELYSCVKSGETLTTISSHIVCPPFQESNCKTETIQTAANGCCKTCVE--
Zebrafish : .........-...VNH..QE.A.LPTSNPG.DKI..S.VNGQFI..KYT.Q....NI...QPG...LS.....HV..D--
Chicken   : ....K...HTSHS.NL..NP.EFINDPYN..T....TSIKNQ.I.S..E.T..A.N.ES..PG..TFLP........P-L
Human     : .E......KRP.NQHV..KP.DFK.DPKN..TF.....IHNQ.I.SV.N.T..N.DA.I.IPG..TFMP.......TPRN

GSB       : -KEKACKLVTMKTHITHKSCQSYQEVQMPYCEGSCNTFTKYSEAAASMQHSCSCCKETRFSNRTIDLHCLNGEVVPYTYI
Zebrafish : -QI.G.Q.Q...DF.K.ND...EKK.D.A..G.D.......ANPGL.---.....QA..S.......A....D..NH...
Chicken   : DSPTP.S.RER.DF.VY.N.R.L.R.V.TE.....G...L..VE.S...........VET.M.E.E.K.PS.HS.THK..
Human     : ETRVP.ST.P.T.E..YAG.T--KT.L.NH.S...G..VM..AK.QA.D........E.T.Q.E.V.S.P..GS.TH..T

GSB       : HVEECGCGHTECTRAVG--------------QSARRRRSFTLA
Zebrafish : .....S.SR.K.HE.EVSQ---------TLA..S.....VRFP
Chicken   : Y..S...QD...IVPESSESQSTEENDESTQNHK..AI.L.SK
Human     : ...S.Q.QD.V.GLPT.-----------TSRRAR.SP.HLGSG
```

Mucin 2-like:

```
GSB         : QQLDLLLQPHNSNDEMELCVAVFLALSVGSVTDVQARLVRNHVSSICSTWGREHFKTFDGDVYQFPGTCEYNLASDCHET
Zebrafish   : ------..WRT.T-----VCM.L....-.IQV.SKKVSPS...N....M..NF..............M.....V...QSL
Turkey      : ------.GLRAAS-----L.V......--NA.E.RKGRT...GHY......NN..............M....FV...QD.
Chicken     : ------.GLRAAS-----LFM......--NT.E.RKGRT...GHY......NN..............M....FV...QD.

GSB         : FQEFSVHMKRKEHDGN-PTVSYVVVTINDLSFHLGKSLVTVNGIPVKLPYYNAGVQVERNVIYLKLQSKVGLVVMWNGDD
Zebrafish   : IR....Y...T.RSTG-.K..R.S......AIE.TENQ.N..EAK.T..VHVS..L..E.T..T..Y.....T....K..
Turkey      : .R......Q.ALNSN.H.E.Q......T.F.VY.KPK.AV...RI..T...SS..L..S.D..T..YA.........QE.
Chicken     : .R......Q.ALNSN.H.E.Q......T.F.VY.KPK.AV...RI..T...SS..L..S.D..T..YA.........QE.

GSB         : AVMVEVDRDYANRTCGLCGDFNGVSVYNEFIHNDRK--------ISSIEFGNKHKVHRPNDECEDPYEEEEESAEASLES
Zebrafish   : .......SK.S.............P......QSG.T--------.GYT....M...PN.TH.......NVD.--.NV.DQ
Turkey      : .......SK.G.Q...........P......SGVAS--------YN..T...LQ..S.........D.------TQA.P.
Chicken     : .......SK.G.Q...........P......SGGESYMLCTCILYN..T...LQ..S.........D.------TRA.P.

GSB         : CKEFQTTCDQILRSEPWSSCTNQINPEPYIQACVQDMCGCTNSTSD-FCLCSTLSEFSRQCSHAGGQPPNWRTPQFCAKQ
Zebrafish   : .EK.RAD.AD..ED.K.....WV....A..K..TN...NRQPEDE.TTA..A..............N..A...AK..NV.
Turkey      : .N.HRDE.EK..T.SA.AD.HSR..L.M........K.S.K-GNE.S...................R.GE...EN..Y.T
Chicken     : .N.HREE.EK..T.SA.AD.HSR..L.M........K.A.K-GNE.S...................R.GE...EN..Y.T

GSB         : CPFNMVYEESGSPCMDTCTHQDTSSLCEDHKMDGCFCPSGTVFDDISMRGCIAQSECQCK-HNKIYNSGEVYRQNSEECT
Zebrafish   : ......HS............K..NA...E.N.......P........DT...PAEK....-.....S....L.KSE...Y
Turkey      : ..G....R..S.........LEV.....E.Y.......E........EN...PV...Y..L.G.G.SP..TISNEC....
Chicken     : ..G....R..S.........LEV.....E.Y.......E........EN...PV...S..L.G.G.SP..TITNEC....

GSB         : CFEGQWACKSLPTPATCAVEEGSHVTTFDGKTYTFHGECYYTLAKVESKDDASPKFTPLAQLAPCANQKFDTCLKALKIV
Zebrafish   : .Q..S.V.M...G.GL........F......E.....D.N.L.S----..CEES..II.G..V..FTHET.....S.V..
Turkey      : .DS.R.T..D..C.G..S..G..........K.....D...V..----.SAVNNTHAL.......GSTDGQ....T.V..
Chicken     : .DS.R.T..D..C.G..S..G....K.....K.....D...V..----.SAVNDTHAL.......GSSDGQ....T.V..

GSB         : LNNDRNNVLMFTSDGTVKQN
Zebrafish   : F....K.P.FIKA....QH.
Turkey      : ..-..K.....R.....SL.
Chicken     : ..-..K.....R.....SL.
```

Mucin 13:

```
GSB   : MAQKFKLLFLLSLVVACLVIPDNPVAAATIDAPVTTDPSTTNLPVSTKPSATSE-----PSVSTKPSATSEPSVSTKPSA
Rat   : -MSQSSGGTSTPTTT.TQPTSTSTQTPG.TQLLS..STP..TATQP.ST.TQ.PGTTQL..T..T..T.ATQPTX.STQT
Dog   : ----M.G.I..A...SISS.SHSK.NCRH..SYHN.YHH.NSYHH.NSYHHINS----YHQTN.YHHTN.YHH.N.YHQT
Human : ----M.A.IH....ALLS-.NTATNQGN.A..VT..ETA..GPT.AAAD.TE.N----F.ETA.-T..N.PSFP.ATSP.

GSB   : T------------SEPSVSTKPSATSEPSVSTKPSATSEPS------------------------------------VST
Rat   : PGTTQLPGTTSTP.TTATQPTS.SFQT.GT.QL..S..T..TTATQPTSTASQTPGTTQPPGGASSPTTTVTQPTGSS.Q
Dog   : N---SYHHTNSYHHIN.YHQTN.YHHTN.YHHIN.YHQTN.YHHTN-----------------SYHHINSYHQTNSYT.P
Human : P---PIISTHSSS.I..PAPPIIS.HSS.TIPI...ADSE.TTNVN-----------------SLATSDIITAS----.P

GSB   : KPTATSDPSVSTKPSVTVDPSVSATP-----TSSEVTDAETSAPSLTTPNP-DSCDSNPCGPGSTCEARAYPNYVCLCQA
Rat   : T.GT.QP.GGA.T..T..TQPTGS.SQTSGT.QPPGGASS..VT.S..TGSN.P......KSPA..VK-L.DS.F...LE
Dog   : T..I..T.ASRPS..T..T..T.S..----T.KPTSPIST.LTQG.VEEG.SSP.QGA..KGD...VS-LNST.F...FF
Human : NDGLI.MVPSE.QSNNE.S..TEDNQ----S.GPPTGT.LLETS..N.TG.S.P.QD...ADN.L.VK-LHNTSF...LE

GSB   : GDIYNHVSNSCQNAKVFPGNLFAPELPYEPEMSDPKSTEFLEAAEKIMSQLQEVFKANTTYTGSTVLKLEPF--TTARVW
Rat   : .YY..N-.S..VKGTT...-EIGMS.NETTD.E.KN.VN.QTLHSS..KFF.NT..K-.D.GQ.V....SKDSLM.S.SV
Dog   : .YY..--.S..KKG.....-TIKV..SEVSG.G.EN.LA.Q.LHN...EFFGGT..N-FD.GQ.V.DG.SIR--P....E
Human : .YY..--.S..KKG.....-KISVT.SETFDPEEKH.MA.QDLHSE.T.LFKD..GT-.V.GQ.V..T.STS--L.P.SE

GSB   : SREGNPHIKATVEISFTATADVTTDGVLEEIKSYVDTCSVNCLLNGATFISETLCAKN-------------ACDAATTR-
Rat   : M.AATQT.YV..VNM.GENTKEDEES.ASV..EA.K.DN-.VERYFQQDRCDYYGCVK-SGSNVCRNGLQCT.KPGLE.L
Dog   : M.A.GTG.EV..LNI.AE.TKENEGT...L.TKA.ENNKN.I.GYSNQS.CDYFNCEKENAQDKCDNGLLCK.KPGFE.P
Human : M.AD.KF.NV..VTILAE.TSDNEKT.T.K.NKA.R.S.S.F.NYDL.LRCDYYGCN--QTADDCLNGLACD.KSDLQ.P

GSB   : ------CLSTDGSFKCDC-----RESYVPTAFSDRMCIACPSGQGVDKGKCVKCSFGRSGLNCSESWQLTLVIVASVLGA
Rat   : NPQVPF..A--P.CSEP.SAEK-..LC.KKDNGAME.GCMAGYR-KAN...EE.P..Y.....KDQ...I.T..G..A..
Dog   : NPQIPM..A.AP.CPDT.NANNN..CL.NKDN.GVE..CLSGYKED.H.I.Q..G..YG....EDQ...I.T..G..G..
Human : NPQSPF..A.SLKCPDA.NAQH-..CL.KKSGGAPE.ACV.GY.EDAN.N.Q..A..Y.....KDK...I.T..G..A.I

GSB   : LLLITLIILPIVALKPSKKRS--------------------------------PKKNKNSRHSGALRQPHSR--------
Rat   : F...L...FI.SMRSKN....GEEQNLIEDDFHNLRMR-PTGFSNFGADTSIF..VKTGVP--SQTSN.YANHRSMPRPD
Dog   : ...GL..A.IC.TRSKN.D.YIEEQNLIENDFQNLRLQQTTGFSNLGADGSIF..VRA.IS--.QPQN.YANHRSMPRPD
Human : ...SM..A.I.T.RSNN.T.HIEEENLIDEDFQNLKLR-STGFTNLGAEGSVF..VRITASRDSQ.QN.Y.SHSSMPRPD

GSB   : -
Rat   : Y
Dog   : Y
Human : Y
```

Mucin 18:

```
GSB        : -----------------------------------MEDRVEAYLGEEARINCMFTSAEGVGALRIHWFFVTRSGEKKQIY
Tilapia    : -----------------------------------......VMA.DT.Q.T.....D....GTT.Q....LPNKK.QR..
Zebrafish  : MDNFRNMALQNTSLLLIVLQICALAWQAWAQVDLR...T..V..DDS.E.P....FT.APMM.M.Q..VRE.D.V.VR.S
Human      : ----MGLPRLVCAFLLAACCCCPRVAGVPGEAEQPAPEL..VE..ST.L.K.GL.QS..N-LSH.D..S.H.--...T.I

GSB        : YLESTMKYADRGTQFTDRVNGTISPGTAVLTISDVHIEDEVEFICHIKSLT-EGREGRTKLKVFDAPAFPSIEGIQTGIS
Tilapia    : NK.PMHEA.EQN.P.....SLN..GTA...V.R..G.K.N.....V.....P.EA....Q.Q..KS.T.....A......
Zebrafish  : .SDL..QKV.EN.T.....SVRSNSDGET...Q..K.S..R..F.Q.SGF.AGSD.R..L.....P.EP.V...AL....
Human      : .RVRQGQGQSEPG..EQ..SLQ--DRG.T.A..Q.TP...RI...QG.RPR--SQ.Y.IQ....K..EE.N..VNPL..P

GSB        : VNEVNPSKIGTCEVKNGFPKPNITWYRNNTPLRPAQDEVEITLSVTVESSGLFSVRSELRMKVTKEDKDDQFYCEVTYFV
Tilapia    : ...E.........S.................M...ITP...D.KS...T.........Q.......K.....AK.....L...
Zebrafish  : .SGESTA..A...T...............HI..H..NGL.N.VTL..R.........Q.D.HY.........AH.S.......
Human      : ..SKE.EE.A..VG.....I.Q.I....GR...EEK.R.H.QS.Q...........Q.I..AQ.V.....A......N.R.

GSB        : PGGTRMTETNRINITVYYPSTAVSVWVESPKGKIKEGDSIMLQCRGNGNTPSSFLSFKHG------EEELPSEQDTLVLH
Tilapia    : ..A.....QH........P.E.N.............E.R.EN...Y...IT.I..Q------NSGYFV.N.K..QT
Zebrafish  : ..AV.T...KG..........S.E..K...Q.L.......E.H.Q....P.API.FN.EQS-----DV..D.Y.GL...K
Human      : .S.NH.K..RE.T.P.....EK.W.E..P-V.M.....R.E.R.LA...P.PH.SIS.QNPSTREA...TTNDNGV...E

GSB        : NATRLDSGVYLCISTDTDTF--EEIAGNTTLFVHYLDPAVVIPEDTIVPQGQD-LKATCNALSSVDTHTAWFKDGE--EI
Tilapia    : .V....G...E.TFME..N.--....S.S.K.L.N..ES...T.K...TVME.EE...........Q.DIT....EK--L.
Zebrafish  : EV.........E.HAL.L.AVNND..LDTLQ.T...........K..E.MLKGES.T.........E.S.V...K.L--..
Human      : P.R.EH..R.E.QGL.L..M-ISL.SEPQE.L.N..SDVR.S.AAPER-.EGSS.TL..E.E..Q.LEFQ.L.EETGQV.

GSB        : SKGNTLIVKDATFDTAGTYECVVTVPEIEGMETRGTLYVNVQGQPEIIKPDN-TEMVQQVETTVSLHCKVRGFPAPRVEW
Tilapia    : ......S..S...E...M.K...........K.SS..Q...K.S....G.G--V.E..TY....N.N.SA......T.M.
Zebrafish  : G..HI.Q.Q........R.D.E....S.P..L.S...H.I...A...KDAERDF....K.GAW.N.T.E.....R.A.T.
Human      : E..PV.Q.H.LKREAG.G.R..A....S.P..NRTQL.N.A.F.P.W.AFKER--K.WVKENMV.N.S.EAS.H.R.T.S.

GSB        : TTA--DGKVLDETSREVTDEGVQSTVDVKVTSDITVFCNATNDFGTDALTFDIKATTHTTAATTTAETTTTITT------
Tilapia    : ..S--...N..ALQT.T-ED.A..V.TF....S.K...........KV.E.....ILN.ILT.I.SMP.I.T..RSTTTI
Zebrafish  : .ITGSQSQSWR.VD.KE.EDR.H.V.T......TVAI..S.....IETKI....SIP--FLQ.P..RK..----------
Human      : NVN---.TASE.DQDPQRVLSTLNV.VTPELLETG.E.T....L.K.TSILF.ELVNL..LTPD.NT..GLS..------

GSB        : ----------TTTSPASTTTISSTTVKPKT--------VNPPKK--KVKKESSGGVIIAVIIICILLLAILGSVLYFLYK
Tilapia    : SNDTVVHTTP....TT..NA..DD..TQN.STNYVNDP.QT.T.SPN.....K.-F.......A...............
Zebrafish  : -----------------------------------------------VDN..-...V....S..........F.....
Human      : -------------.ASPH.RAN....E.KLP------------------EP..R.-...VA............A......

GSB        : KGK-ICGRSGKQDLTKEKSNKDNIVVEMKSDNT-EEAVLLG-VNGEKQPPNDQ---------
Tilapia    : ...-...........F..R..S...............-.........-......Q....---------
Zebrafish  : ...L........E.................AKK.-..S...KG.....K.....NSTLRS---
Human      : ...LP.R.....E..LPP.R.SE........KLP..MG..QGSS.D.RA.G..GEKYIDLRH
```

Mucin 19:

```
GSB       : ---------------FLLLACSKESSDTMTSQRWMLAVWLVLSSVLGTGESVATTQTQKYTCRTFGSGVVQPFNDSVFYV
Tilapia   : ----------------------------..P.QR..S.CF..V..T---------------............KGLG...
Mole rat  : --------------------EKGSHIPEA.PKYSETN-------------------EIIGEAS..DR.TYKA..GH..SF
Cow       : GLSGSASVEDELKGFASDASS.GGNIW.SN.GSGEGNKGEAGLGTS.QNV.DE.GV.STGEAS...K.AYKA..GR..SF

GSB       : RSNCPFTLT-HFTHNRVECDITTRRGDNGLLVQVEIIINKVRTVLQNGSIQVERKSVSLPYDHTYQHIFHYGIYTKLRSP
Tilapia   : .........-R.......Y...IQ...........T.............L.................Q.........S
Mole rat  : ..S.T..FCR.CVESGGDF..EI..N.-SG.EK.K....SNDIS.L.D..L.NGER.Q....SKLI..KKL.K.NV...R
Cow       : E.S.T..FCR.CVESGGDF..EI..N..SE.EK.T....NNDVS.FGDI.L.NGE..Q....NKLI..KK..EHNV.N.R

GSB       : LPPLSVTWHNGPGGIDTLWVELEQELSTDMTGLCGKHNGTGN---QKVVMESVLADDTCQTRDPTSVGSPECGTFFSHTL
Tilapia   : .L.....RSV..................G.......D.A.S.N--.Q..T..T.TQE.......VF.TN.V.RK...Y..
Mole rat  : RGI...M.DKN----...S.T.HKRYE.--C....NF.S.TGQDINEH..N.K.PG.CPSAVSK.YEVCEDGVQ.CNNI.
Cow       : RGI...M.DKN----.K.S.T.HK.YP.--C....NF.S.PGDDINEH.AD.K.P..CSKAVSK.YEVCEDGVQ.CNKI.

GSB       : ECLQARVRQYLDLCEKN------IYKYEMSKGVTCSFFKEIVQQCENT-SRVWDIWR--SVTKCDKPDCPGDLIYVEQGP
Tilapia   : ....V.TPH..Q...A.------F.S..K..Y.G.A.....ALH.G.N-.Y..QK..--A....AE.T.......K...D
Mole rat  : GTYFE.CEKVAT.SSGYKTICINE.CQRGE.SS..DT.S..SRL.ASDGPG..ES.LDD.NVT.E..T..EKQ..K.C.L
Cow       : GTYFE.CGKVST.SSDYKMICIDE.CQSRD.TS..DT.S..SRL.ASDGPGT.ES..DDPDVV.E..I..EKH..K.C..

GSB       : AFVPSCTNPNPRFSNQDYISTCVCPEGKVLNDQADGSHCVSVSSSPCVFAGKSYSTGDMRSTKC-QLCICDSGKWRCSEN
Tilapia   : ...........S..G.AIT.....K.....H.EDL.....P.C....S....L.....K...-.A....G...H....
Mole rat  : SNPA....VA.-.Q.SEC..G.T.S..Y....IGEKGK..LKE.C..ESN..V.NP.E..EGP.GSQ.T.QDA..S..DG
Cow       : SNPA....VA.-.Q.TEC..G.T....Y....IGEKGR..LK.DC..ESN..V.Q..E..EGS.GS..T.QEA..S..KT

GSB       : ICPARCVIEGQHVTTFDGKEYVLPGKCTYVALQGFNWTIKIEFSVKDASLKTVAFLLFQEKYTVSQGVVKIGEEE-----
Tilapia   : S..GG.H....F.......K...........S..L....N....G.H....R.ILQ....M..F.HN...FED..-----
Mole rat  : ...G..K...SLF....DNK.NH..N.H....HNE....S..LRPCPN.QTGTCLNSVTLLLNS.VS...YVFNSDGKVT
Cow       : ...G..K...SL......VK.NH..N.H....HDK....S..LRPCPSGQSGTCLNSVTLLLNS.VQ.D.YVFNRDGTVT

GSB       : -----ITELHQSDNVLVFWQSSMYIHFHTSLGMKIEVQMSPEIQLYITPPANHTGMISGLCGNNNNDTTDDFTTTSGIIE
Tilapia   : -----.........RA..........EV...F....................G..............................
Mole rat  : KDMVR.KDYYY..E.Q..KA..S..QVE.YNH......TV.V......M.P.EFTDTV....SY..KAE...M..QN...
Cow       : NDKFGNLGYYY..K.Q..NA..S..QAE.YFHG......F.V......M.P.QFTDTV....SH..RAE...M..QN...

GSB       : NSAQPFAQSWSVGDCAVDIPDTCINKDNEIFADEK
Tilapia   : ..P....L.....P.TP...P....T.........
Mole rat  : K.S.A..N..E.MP.PKGT.A...SIEK.K..ENN
Cow       : K.S.A..S..E.MP.PKASTA...SIEK.R..ERH
```
