# Supplementary material for: Mucins as Diagnostic and Prognostic Biomarkers in a Fish-Parasite Model: Transcriptional and Functional Analysis
Source: PLoS One. 2013 Jun 12;8(6):e65457. doi: 10.1371/journal.pone.0065457 (PMC3680472; doi:10.1371/journal.pone.0065457)
Supplement: Table S1 — Fish Oil (FO) and 66% Vegetable Oil (66 VO) diet ingredients. (DOCX) [file pone.0065457.s002.docx]

# **Supporting information**

**Table S1.** Fish Oil (FO) and 66% Vegetable Oil (66VO) diet ingredients.

| Ingredient (%) | FO | 66VO |
| --- | --- | --- |
| Fish meal (CP 70%) ^1^ | 15 | 15 |
| CPSP 90 ^2^ | 5 | 5 |
| Corn gluten | 40 | 40 |
| Soybean meal | 14.3 | 14.3 |
| Extruded wheat | 4 | 4 |
| Fish oil ^3^ | 15.15 | 5.15 |
| Rapeseed oil | 0 | 1.7 |
| Linseed oil | 0 | 5.8 |
| Palm oil | 0 | 2.5 |
| Soya lecithin | 1 | 1 |
| Binder | 1 | 1 |
| Mineral premix ^4^ | 1 | 1 |
| Vitamin premix ^5^ | 1 | 1 |
| CaHPO_4_.2H_2_O (18%P) | 2 | 2 |
| L-Lys | 0.55 | 0.55 |
|  |  |  |
| Proximate composition |  |  |
| Dry matter (DM, %) | 93.13 | 92.77 |
| Protein (% DM) | 53.2 | 52.62 |
| Fat (% DM) | 21.09 | 20.99 |
| Ash (% DM) | 6.52 | 6.57 |

^1^Fish meal (Scandinavian LT)

^2^Fish soluble protein concentrate (Sopropêche, France)

^3^Fish oil (Sopropêche, France)

^4^Supplied the following (mg / kg diet, except as noted): calcium carbonate (40% Ca) 2.15 g, magnesium hydroxide (60% Mg) 1.24 g, potassium chloride 0.9 g, ferric citrate 0.2 g, potassium iodine 4 mg, sodium chloride 0.4 g, calcium hydrogen phosphate 50 g, copper sulphate 0.3, zinc sulphate 40, cobalt sulphate 2, manganese sulphate 30, sodium selenite 0.3.

^5^Supplied the following (mg / kg diet): retinyl acetate 2.58, DL-cholecalciferol 0.037, DL-α tocopheryl acetate 30, menadione sodium bisulphite 2.5, thiamin 7.5, riboflavin 15, pyridoxine 7.5, nicotinic acid 87.5, folic acid 2.5, calcium pantothenate 2.5, vitamin B_12_ 0.025, ascorbic acid 250, inositol 500, biotin 1.25 and choline chloride 500.
