# Supplementary material for: Mucins as Diagnostic and Prognostic Biomarkers in a Fish-Parasite Model: Transcriptional and Functional Analysis
Source: PLoS One. 2013 Jun 12;8(6):e65457. doi: 10.1371/journal.pone.0065457 (PMC3680472; doi:10.1371/journal.pone.0065457)
Supplement: Table S2 — Relative expression values of gilthead sea bream mucins in all studied tissues. (DOCX) [file pone.0065457.s003.docx]

# Supporting information

**Table S2.** Relative expression values of gilthead sea bream mucins in all studied tissues. For each tissue, different letters

stand for statistically significant differences (P<0.05, one-way ANOVA) among mucins.

| Tissue | I-Muc | Muc2 | Muc2-like | Muc13 | Muc18 | Muc19 |
| --- | --- | --- | --- | --- | --- | --- |
| Gills | 0.71 ± 0.02^c^ | 0.09 ± 0.01^a^ | 0.08 ± 0.01^a^ | 0.11 ±0.02^b^ | 54.90 ± 1.59^d^ | 0.18 ± 0.01^bc^ |
| Esophagous | 195.64 ± 13.51^d^ | 0.44 ± 0.03^a^ | 0.85 ± 0.03^a^ | 2.35 ± 0.11^b^ | 113.56 ± 3.97^c^ | 3600.58 ± 336.82^e^ |
| Stomach | 42.91 ± 2.37^b^ | 1.00 ± 0.19^a^ | 0.92 ± 0.16^a^ | 0.94 ± 0.13^a^ | 59.15 ± 2.46^b^ | 1.67 ± 0.22^a^ |
| Skin | 4.06 ± 0.14^b^ | 0.93 ± 0.06^a^ | 0.92 ± 0.07^a^ | 1.58 ± 0.17^ab^ | 180.47 ± 2.95^c^ | 0.53 ± 0.06^a^ |
| A. Intestine | 5.13 ± 0.16^b^ | 1449.26 ± 26.29^d^ | 1481.46 ± 44.57^d^ | 2407.70 ± 52.36^e^ | 50.56 ± 1.82^c^ | 0.52 ± 0.05^a^ |
| M. Intestine | 4.23 ± 0.23^b^ | 1111.80 ± 34.23^d^ | 1142.07 ± 33.27^d^ | 2566.91 ± 107.04^e^ | 55.78 ± 1.68^c^ | 0.39 ± 0.06^a^ |
| P. Intestine | 1806.75 ± 75.92^d^ | 20.68 ± 20.78^c^ | 961.60 ± 23.41^c^ | 2671.51 ± 73.05^e^ | 82.18 ± 3.30^b^ | 0.29 ± 0.03^a^ |
